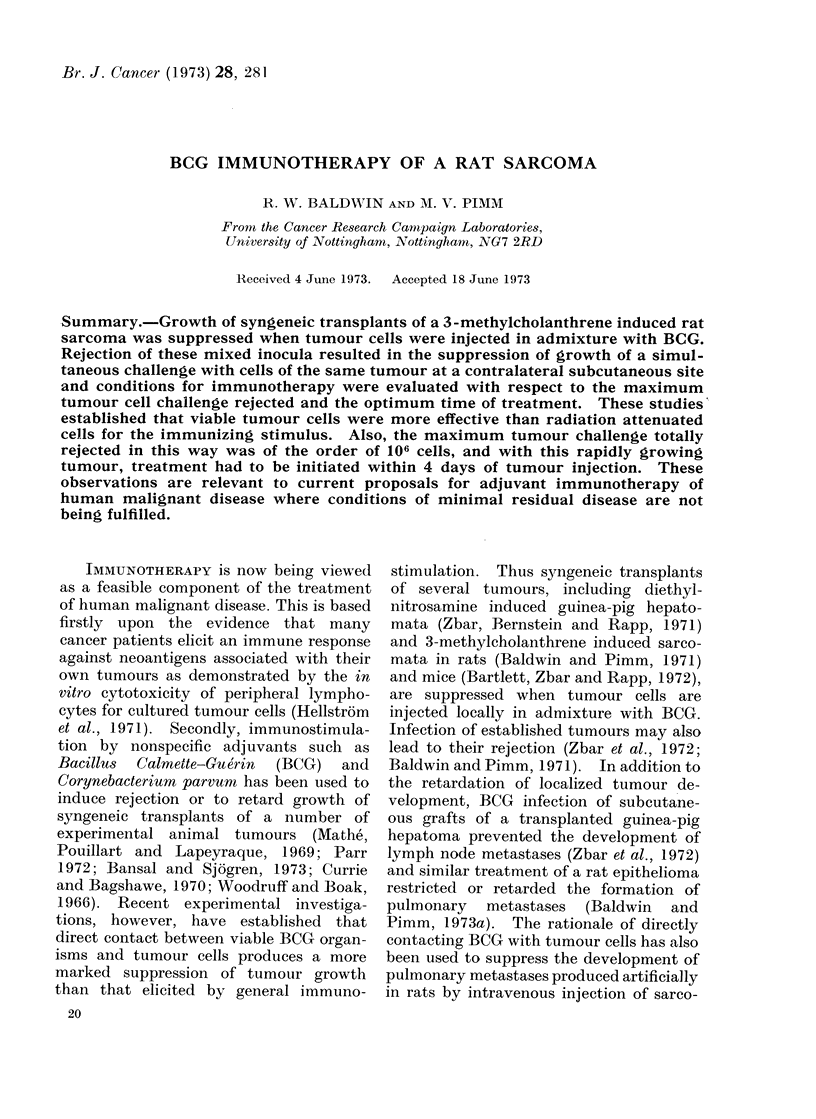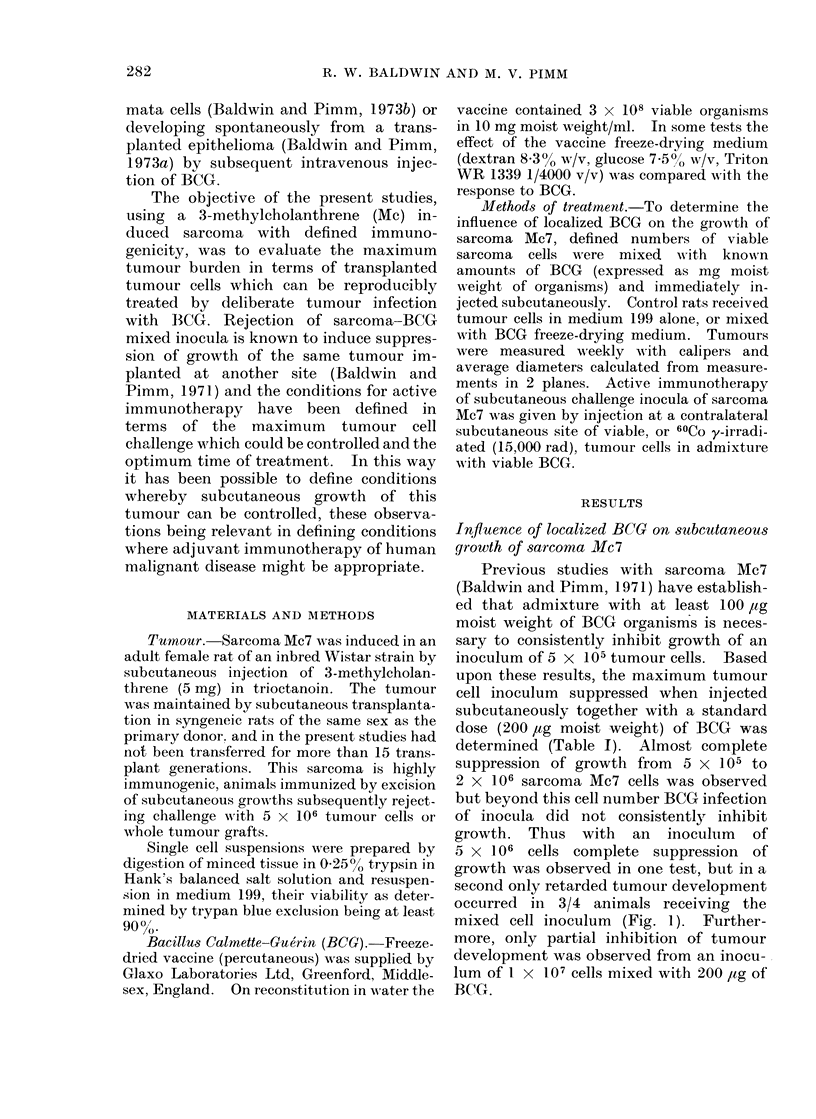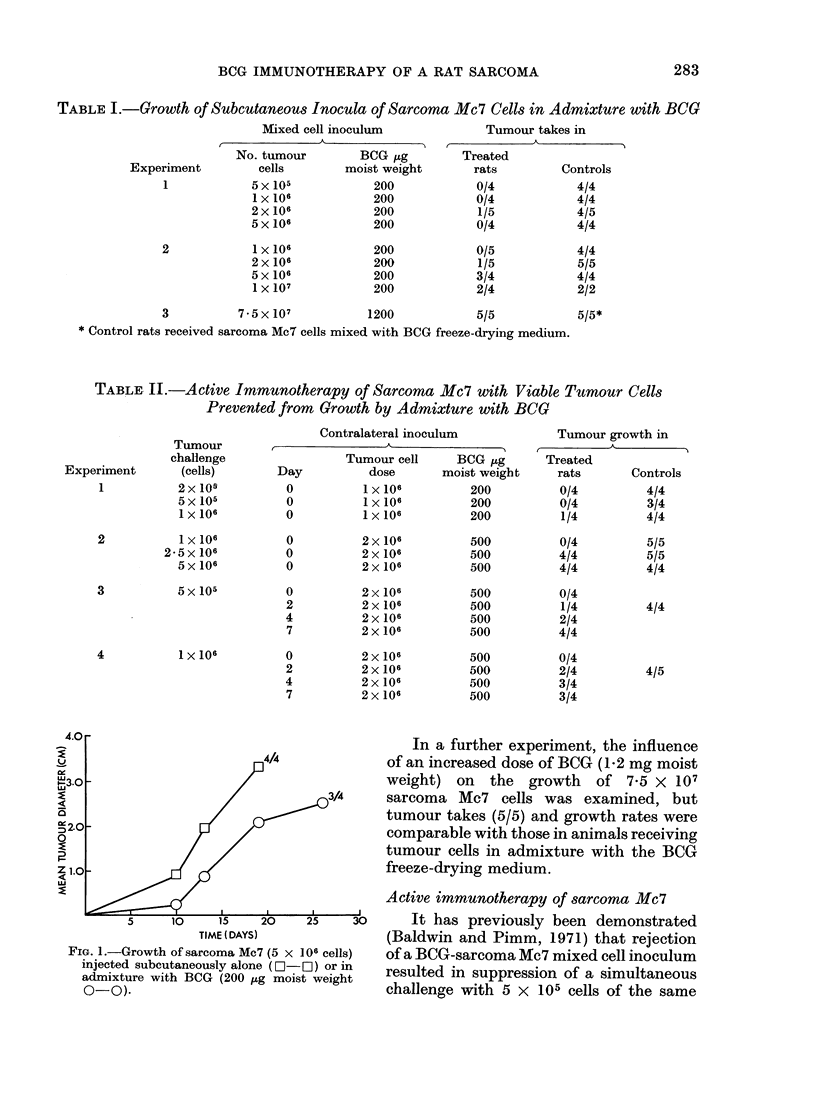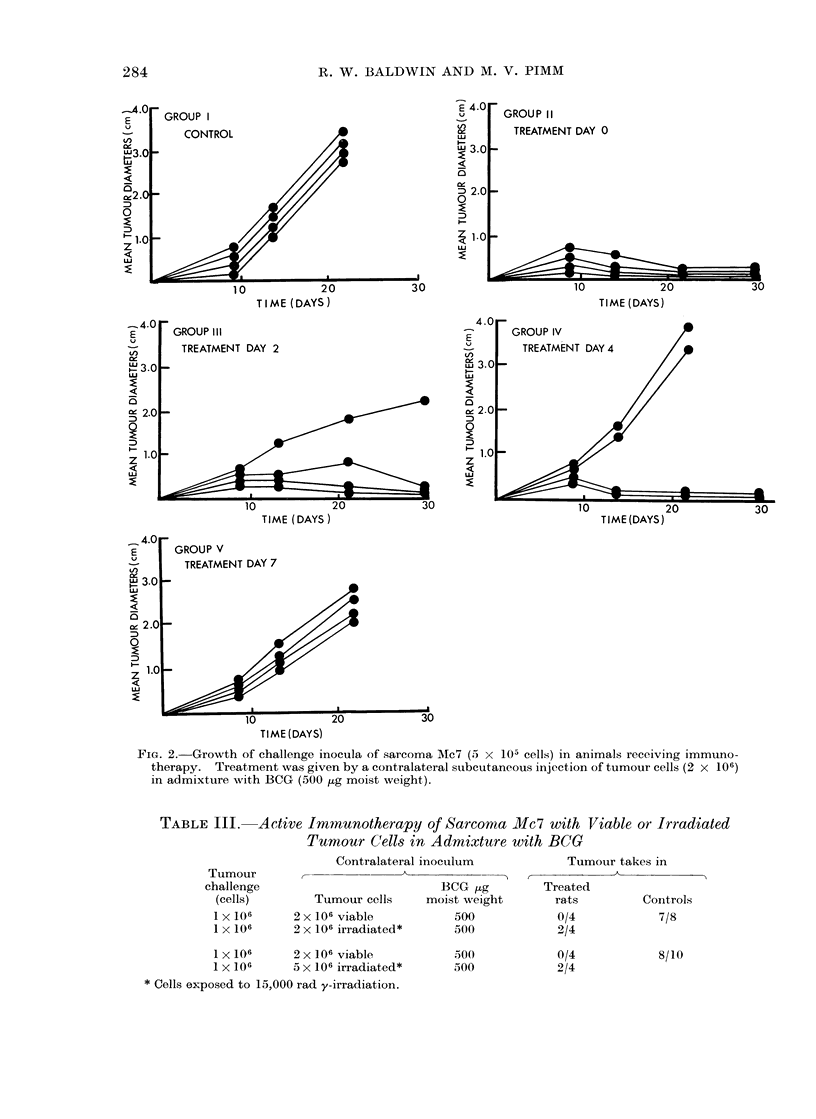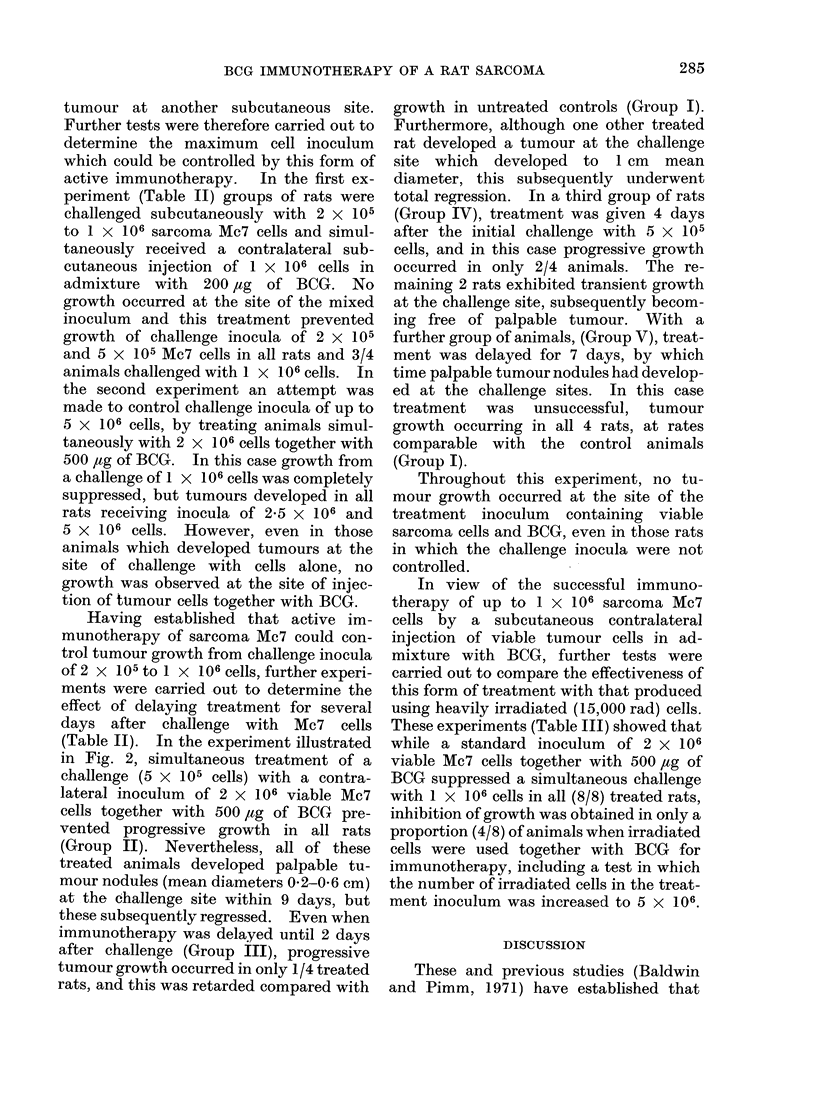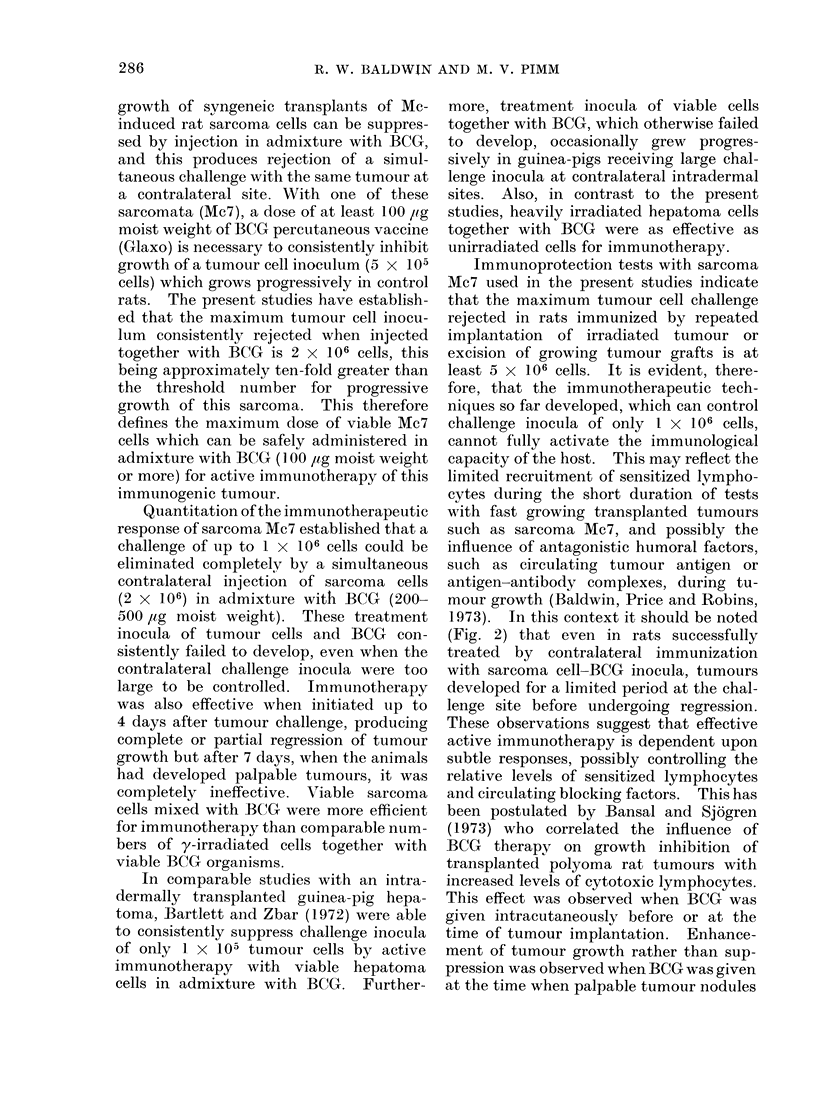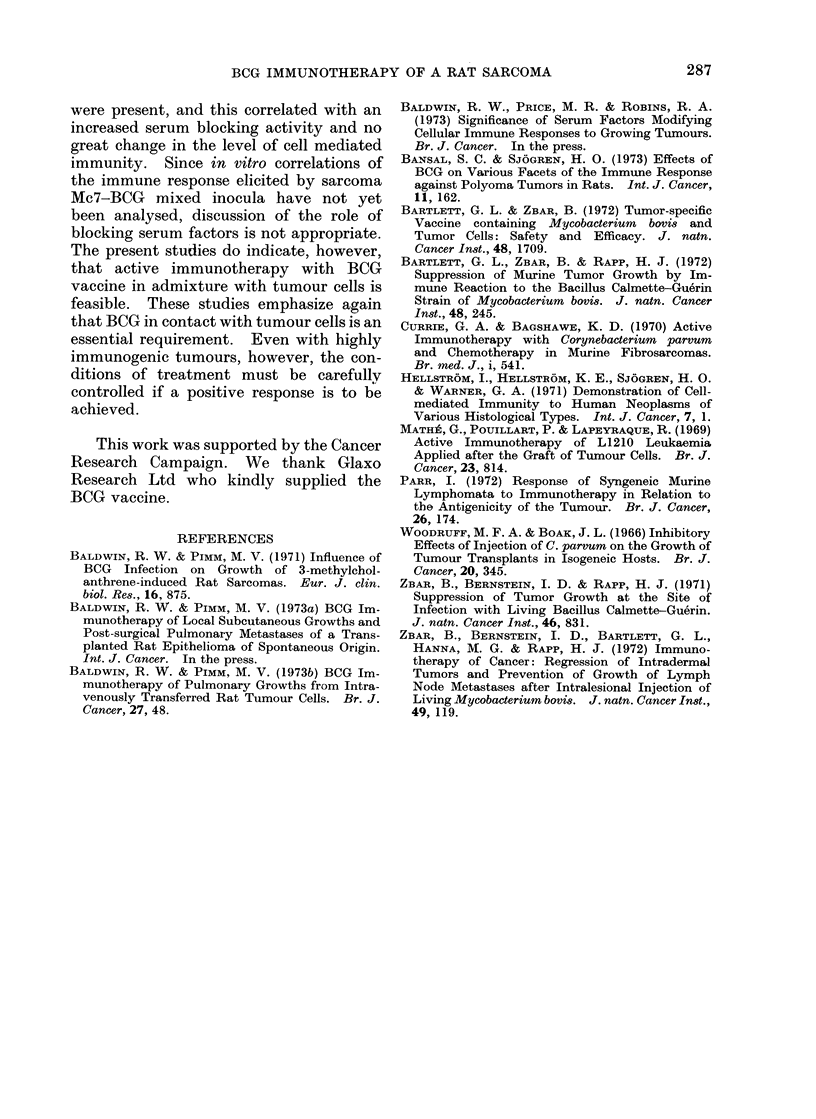# BCG Immunotherapy of a Rat Sarcoma

**DOI:** 10.1038/bjc.1973.149

**Published:** 1973-10

**Authors:** R. W. Baldwin, M. V. Pimm

## Abstract

Growth of syngeneic transplants of a 3-methylcholanthrene induced rat sarcoma was suppressed when tumour cells were injected in admixture with BCG. Rejection of these mixed inocula resulted in the suppression of growth of a simultaneous challenge with cells of the same tumour at a contralateral subcutaneous site and conditions for immunotherapy were evaluated with respect to the maximum tumour cell challenge rejected and the optimum time of treatment. These studies established that viable tumour cells were more effective than radiation attenuated cells for the immunizing stimulus. Also, the maximum tumour challenge totally rejected in this way was of the order of 10^6^ cells, and with this rapidly growing tumour, treatment had to be initiated within 4 days of tumour injection. These observations are relevant to current proposals for adjuvant immunotherapy of human malignant disease where conditions of minimal residual disease are not being fulfilled.


*Br. J. Cancer* (1973) **28**, 281

### BCG IMMUNOTHERAPY OF A RAT SARCOMA

R. W. BALDWIN AND M. V. PIMM

*From the Cancer Research Campaign Laboratories, University of Nottingham, Nottingham, NG7 2RD*

Received 4 June 1973. Accepted 18 June 1973

**Summary.—Growth of syngeneic transplants of a 3-methylcholanthrene induced rat sarcoma was suppressed when tumour cells were injected in admixture with BCG. Rejection of these mixed inocula resulted in the suppression of growth of a simultaneous challenge with cells of the same tumour at a contralateral subcutaneous site and conditions for immunotherapy were evaluated with respect to the maximum tumour cell challenge rejected and the optimum time of treatment. These studies established that viable tumour cells were more effective than radiation attenuated cells for the immunizing stimulus. Also, the maximum tumour challenge totally rejected in this way was of the order of $10^6$ cells, and with this rapidly growing tumour, treatment had to be initiated within 4 days of tumour injection. These observations are relevant to current proposals for adjuvant immunotherapy of human malignant disease where conditions of minimal residual disease are not being fulfilled.**

IMMUNOTHERAPY is now being viewed as a feasible component of the treatment of human malignant disease. This is based firstly upon the evidence that many cancer patients elicit an immune response against neoantigens associated with their own tumours as demonstrated by the *in vitro* cytotoxicity of peripheral lymphocytes for cultured tumour cells (Hellström *et al.*, 1971). Secondly, immunostimulation by nonspecific adjuvants such as *Bacillus Calmette–Guérin* (BCG) and *Corynebacterium parvum* has been used to induce rejection or to retard growth of syngeneic transplants of a number of experimental animal tumours (Mathé, Pouillart and Lapeyraque, 1969; Parr 1972; Bansal and Sjögren, 1973; Currie and Bagshawe, 1970; Woodruff and Boak, 1966). Recent experimental investigations, however, have established that direct contact between viable BCG organisms and tumour cells produces a more marked suppression of tumour growth than that elicited by general immunostimulation. Thus syngeneic transplants of several tumours, including diethylnitrosamine induced guinea-pig hepatomata (Zbar, Bernstein and Rapp, 1971) and 3-methylcholanthrene induced sarcomata in rats (Baldwin and Pimm, 1971) and mice (Bartlett, Zbar and Rapp, 1972), are suppressed when tumour cells are injected locally in admixture with BCG. Infection of established tumours may also lead to their rejection (Zbar *et al.*, 1972; Baldwin and Pimm, 1971). In addition to the retardation of localized tumour development, BCG infection of subcutaneous grafts of a transplanted guinea-pig hepatoma prevented the development of lymph node metastases (Zbar *et al.*, 1972) and similar treatment of a rat epithelioma restricted or retarded the formation of pulmonary metastases (Baldwin and Pimm, 1973*a*). The rationale of directly contacting BCG with tumour cells has also been used to suppress the development of pulmonary metastases produced artificially in rats by intravenous injection of sarcomata cells (Baldwin and Pimm, 1973*b*) or developing spontaneously from a transplanted epithelioma (Baldwin and Pimm, 1973*a*) by subsequent intravenous injection of BCG.

The objective of the present studies, using a 3-methylcholanthrene (Mc) induced sarcoma with defined immunogenicity, was to evaluate the maximum tumour burden in terms of transplanted tumour cells which can be reproducibly treated by deliberate tumour infection with BCG. Rejection of sarcoma–BCG mixed inocula is known to induce suppression of growth of the same tumour implanted at another site (Baldwin and Pimm, 1971) and the conditions for active immunotherapy have been defined in terms of the maximum tumour cell challenge which could be controlled and the optimum time of treatment. In this way it has been possible to define conditions whereby subcutaneous growth of this tumour can be controlled, these observations being relevant in defining conditions where adjuvant immunotherapy of human malignant disease might be appropriate.

### MATERIALS AND METHODS

*Tumour.*—Sarcoma Mc7 was induced in an adult female rat of an inbred Wistar strain by subcutaneous injection of 3-methylcholanthrene (5 mg) in trioctanoin. The tumour was maintained by subcutaneous transplantation in syngeneic rats of the same sex as the primary donor, and in the present studies had not been transferred for more than 15 transplant generations. This sarcoma is highly immunogenic, animals immunized by excision of subcutaneous growths subsequently rejecting challenge with $5 \times 10^6$ tumour cells or whole tumour grafts.

Single cell suspensions were prepared by digestion of minced tissue in 0·25% trypsin in Hank's balanced salt solution and resuspension in medium 199, their viability as determined by trypan blue exclusion being at least 90%.

*Bacillus Calmette–Guérin* (*BCG*).—Freeze-dried vaccine (percutaneous) was supplied by Glaxo Laboratories Ltd, Greenford, Middlesex, England. On reconstitution in water the vaccine contained $3 \times 10^8$ viable organisms in 10 mg moist weight/ml. In some tests the effect of the vaccine freeze-drying medium (dextran 8·3% w/v, glucose 7·5% w/v, Triton WR 1339 1/4000 v/v) was compared with the response to BCG.

*Methods of treatment.*—To determine the influence of localized BCG on the growth of sarcoma Mc7, defined numbers of viable sarcoma cells were mixed with known amounts of BCG (expressed as mg moist weight of organisms) and immediately injected subcutaneously. Control rats received tumour cells in medium 199 alone, or mixed with BCG freeze-drying medium. Tumours were measured weekly with calipers and average diameters calculated from measurements in 2 planes. Active immunotherapy of subcutaneous challenge inocula of sarcoma Mc7 was given by injection at a contralateral subcutaneous site of viable, or $^{60}Co$ $\gamma$-irradiated (15,000 rad), tumour cells in admixture with viable BCG.

### RESULTS

### *Influence of localized BCG on subcutaneous growth of sarcoma Mc7*

Previous studies with sarcoma Mc7 (Baldwin and Pimm, 1971) have established that admixture with at least 100 $\mu$g moist weight of BCG organisms is necessary to consistently inhibit growth of an inoculum of $5 \times 10^5$ tumour cells. Based upon these results, the maximum tumour cell inoculum suppressed when injected subcutaneously together with a standard dose (200 $\mu$g moist weight) of BCG was determined (Table I). Almost complete suppression of growth from $5 \times 10^5$ to $2 \times 10^6$ sarcoma Mc7 cells was observed but beyond this cell number BCG infection of inocula did not consistently inhibit growth. Thus with an inoculum of $5 \times 10^6$ cells complete suppression of growth was observed in one test, but in a second only retarded tumour development occurred in 3/4 animals receiving the mixed cell inoculum (Fig. 1). Furthermore, only partial inhibition of tumour development was observed from an inoculum of $1 \times 10^7$ cells mixed with 200 $\mu$g of BCG.

TABLE I.—*Growth of Subcutaneous Inocula of Sarcoma Mc7 Cells in Admixture with BCG*

| | Mixed cell inoculum | | Tumour takes in | |
|---|---|---|---|---|
| Experiment | No. tumour cells | BCG μg moist weight | Treated rats | Controls |
| 1 | $5\times10^5$ | 200 | 0/4 | 4/4 |
| | $1\times10^6$ | 200 | 0/4 | 4/4 |
| | $2\times10^6$ | 200 | 1/5 | 4/5 |
| | $5\times10^6$ | 200 | 0/4 | 4/4 |
| 2 | $1\times10^6$ | 200 | 0/5 | 4/4 |
| | $2\times10^6$ | 200 | 1/5 | 5/5 |
| | $5\times10^6$ | 200 | 3/4 | 4/4 |
| | $1\times10^7$ | 200 | 2/4 | 2/2 |
| 3 | $7{\cdot}5\times10^7$ | 1200 | 5/5 | 5/5* |

* Control rats received sarcoma Mc7 cells mixed with BCG freeze-drying medium.

TABLE II.—*Active Immunotherapy of Sarcoma Mc7 with Viable Tumour Cells Prevented from Growth by Admixture with BCG*

| | | Contralateral inoculum | | | Tumour growth in | |
|---|---|---|---|---|---|---|
| Experiment | Tumour challenge (cells) | Day | Tumour cell dose | BCG μg moist weight | Treated rats | Controls |
| 1 | $2\times10^5$ | 0 | $1\times10^6$ | 200 | 0/4 | 4/4 |
| | $5\times10^5$ | 0 | $1\times10^6$ | 200 | 0/4 | 3/4 |
| | $1\times10^6$ | 0 | $1\times10^6$ | 200 | 1/4 | 4/4 |
| 2 | $1\times10^6$ | 0 | $2\times10^6$ | 500 | 0/4 | 5/5 |
| | $2{\cdot}5\times10^6$ | 0 | $2\times10^6$ | 500 | 4/4 | 5/5 |
| | $5\times10^6$ | 0 | $2\times10^6$ | 500 | 4/4 | 4/4 |
| 3 | $5\times10^5$ | 0 | $2\times10^6$ | 500 | 0/4 | |
| | | 2 | $2\times10^6$ | 500 | 1/4 | 4/4 |
| | | 4 | $2\times10^6$ | 500 | 2/4 | |
| | | 7 | $2\times10^6$ | 500 | 4/4 | |
| 4 | $1\times10^6$ | 0 | $2\times10^6$ | 500 | 0/4 | |
| | | 2 | $2\times10^6$ | 500 | 2/4 | 4/5 |
| | | 4 | $2\times10^6$ | 500 | 3/4 | |
| | | 7 | $2\times10^6$ | 500 | 3/4 | |

FIG. 1.—Growth of sarcoma Mc7 ($5 \times 10^6$ cells) injected subcutaneously alone (□—□) or in admixture with BCG (200 μg moist weight ○—○).

In a further experiment, the influence of an increased dose of BCG (1·2 mg moist weight) on the growth of $7{\cdot}5 \times 10^7$ sarcoma Mc7 cells was examined, but tumour takes (5/5) and growth rates were comparable with those in animals receiving tumour cells in admixture with the BCG freeze-drying medium.

*Active immunotherapy of sarcoma Mc7*

It has previously been demonstrated (Baldwin and Pimm, 1971) that rejection of a BCG-sarcoma Mc7 mixed cell inoculum resulted in suppression of a simultaneous challenge with $5 \times 10^5$ cells of the same

FIG. 2.—Growth of challenge inocula of sarcoma Mc7 ($5 \times 10^5$ cells) in animals receiving immunotherapy. Treatment was given by a contralateral subcutaneous injection of tumour cells ($2 \times 10^6$) in admixture with BCG (500 $\mu$g moist weight).

TABLE III.—*Active Immunotherapy of Sarcoma Mc7 with Viable or Irradiated Tumour Cells in Admixture with BCG*

| Tumour challenge (cells) | Contralateral inoculum: Tumour cells | Contralateral inoculum: BCG $\mu$g moist weight | Tumour takes in: Treated rats | Tumour takes in: Controls |
|---|---|---|---|---|
| $1 \times 10^6$ | $2 \times 10^6$ viable | 500 | 0/4 | 7/8 |
| $1 \times 10^6$ | $2 \times 10^6$ irradiated* | 500 | 2/4 | |
| $1 \times 10^6$ | $2 \times 10^6$ viable | 500 | 0/4 | 8/10 |
| $1 \times 10^6$ | $5 \times 10^6$ irradiated* | 500 | 2/4 | |

* Cells exposed to 15,000 rad $\gamma$-irradiation.

tumour at another subcutaneous site. Further tests were therefore carried out to determine the maximum cell inoculum which could be controlled by this form of active immunotherapy. In the first experiment (Table II) groups of rats were challenged subcutaneously with $2 \times 10^5$ to $1 \times 10^6$ sarcoma Mc7 cells and simultaneously received a contralateral subcutaneous injection of $1 \times 10^6$ cells in admixture with 200 $\mu$g of BCG. No growth occurred at the site of the mixed inoculum and this treatment prevented growth of challenge inocula of $2 \times 10^5$ and $5 \times 10^5$ Mc7 cells in all rats and 3/4 animals challenged with $1 \times 10^6$ cells. In the second experiment an attempt was made to control challenge inocula of up to $5 \times 10^6$ cells, by treating animals simultaneously with $2 \times 10^6$ cells together with 500 $\mu$g of BCG. In this case growth from a challenge of $1 \times 10^6$ cells was completely suppressed, but tumours developed in all rats receiving inocula of $2{\cdot}5 \times 10^6$ and $5 \times 10^6$ cells. However, even in those animals which developed tumours at the site of challenge with cells alone, no growth was observed at the site of injection of tumour cells together with BCG.

Having established that active immunotherapy of sarcoma Mc7 could control tumour growth from challenge inocula of $2 \times 10^5$ to $1 \times 10^6$ cells, further experiments were carried out to determine the effect of delaying treatment for several days after challenge with Mc7 cells (Table II). In the experiment illustrated in Fig. 2, simultaneous treatment of a challenge ($5 \times 10^5$ cells) with a contralateral inoculum of $2 \times 10^6$ viable Mc7 cells together with 500 $\mu$g of BCG prevented progressive growth in all rats (Group II). Nevertheless, all of these treated animals developed palpable tumour nodules (mean diameters 0·2–0·6 cm) at the challenge site within 9 days, but these subsequently regressed. Even when immunotherapy was delayed until 2 days after challenge (Group III), progressive tumour growth occurred in only 1/4 treated rats, and this was retarded compared with growth in untreated controls (Group I). Furthermore, although one other treated rat developed a tumour at the challenge site which developed to 1 cm mean diameter, this subsequently underwent total regression. In a third group of rats (Group IV), treatment was given 4 days after the initial challenge with $5 \times 10^5$ cells, and in this case progressive growth occurred in only 2/4 animals. The remaining 2 rats exhibited transient growth at the challenge site, subsequently becoming free of palpable tumour. With a further group of animals, (Group V), treatment was delayed for 7 days, by which time palpable tumour nodules had developed at the challenge sites. In this case treatment was unsuccessful, tumour growth occurring in all 4 rats, at rates comparable with the control animals (Group I).

Throughout this experiment, no tumour growth occurred at the site of the treatment inoculum containing viable sarcoma cells and BCG, even in those rats in which the challenge inocula were not controlled.

In view of the successful immunotherapy of up to $1 \times 10^6$ sarcoma Mc7 cells by a subcutaneous contralateral injection of viable tumour cells in admixture with BCG, further tests were carried out to compare the effectiveness of this form of treatment with that produced using heavily irradiated (15,000 rad) cells. These experiments (Table III) showed that while a standard inoculum of $2 \times 10^6$ viable Mc7 cells together with 500 $\mu$g of BCG suppressed a simultaneous challenge with $1 \times 10^6$ cells in all (8/8) treated rats, inhibition of growth was obtained in only a proportion (4/8) of animals when irradiated cells were used together with BCG for immunotherapy, including a test in which the number of irradiated cells in the treatment inoculum was increased to $5 \times 10^6$.

### DISCUSSION

These and previous studies (Baldwin and Pimm, 1971) have established that growth of syngeneic transplants of Mc-induced rat sarcoma cells can be suppressed by injection in admixture with BCG, and this produces rejection of a simultaneous challenge with the same tumour at a contralateral site. With one of these sarcomata (Mc7), a dose of at least 100 $\mu$g moist weight of BCG percutaneous vaccine (Glaxo) is necessary to consistently inhibit growth of a tumour cell inoculum ($5 \times 10^5$ cells) which grows progressively in control rats. The present studies have established that the maximum tumour cell inoculum consistently rejected when injected together with BCG is $2 \times 10^6$ cells, this being approximately ten-fold greater than the threshold number for progressive growth of this sarcoma. This therefore defines the maximum dose of viable Mc7 cells which can be safely administered in admixture with BCG (100 $\mu$g moist weight or more) for active immunotherapy of this immunogenic tumour.

Quantitation of the immunotherapeutic response of sarcoma Mc7 established that a challenge of up to $1 \times 10^6$ cells could be eliminated completely by a simultaneous contralateral injection of sarcoma cells ($2 \times 10^6$) in admixture with BCG (200–500 $\mu$g moist weight). These treatment inocula of tumour cells and BCG consistently failed to develop, even when the contralateral challenge inocula were too large to be controlled. Immunotherapy was also effective when initiated up to 4 days after tumour challenge, producing complete or partial regression of tumour growth but after 7 days, when the animals had developed palpable tumours, it was completely ineffective. Viable sarcoma cells mixed with BCG were more efficient for immunotherapy than comparable numbers of $\gamma$-irradiated cells together with viable BCG organisms.

In comparable studies with an intradermally transplanted guinea-pig hepatoma, Bartlett and Zbar (1972) were able to consistently suppress challenge inocula of only $1 \times 10^5$ tumour cells by active immunotherapy with viable hepatoma cells in admixture with BCG. Furthermore, treatment inocula of viable cells together with BCG, which otherwise failed to develop, occasionally grew progressively in guinea-pigs receiving large challenge inocula at contralateral intradermal sites. Also, in contrast to the present studies, heavily irradiated hepatoma cells together with BCG were as effective as unirradiated cells for immunotherapy.

Immunoprotection tests with sarcoma Mc7 used in the present studies indicate that the maximum tumour cell challenge rejected in rats immunized by repeated implantation of irradiated tumour or excision of growing tumour grafts is at least $5 \times 10^6$ cells. It is evident, therefore, that the immunotherapeutic techniques so far developed, which can control challenge inocula of only $1 \times 10^6$ cells, cannot fully activate the immunological capacity of the host. This may reflect the limited recruitment of sensitized lymphocytes during the short duration of tests with fast growing transplanted tumours such as sarcoma Mc7, and possibly the influence of antagonistic humoral factors, such as circulating tumour antigen or antigen–antibody complexes, during tumour growth (Baldwin, Price and Robins, 1973). In this context it should be noted (Fig. 2) that even in rats successfully treated by contralateral immunization with sarcoma cell–BCG inocula, tumours developed for a limited period at the challenge site before undergoing regression. These observations suggest that effective active immunotherapy is dependent upon subtle responses, possibly controlling the relative levels of sensitized lymphocytes and circulating blocking factors. This has been postulated by Bansal and Sjögren (1973) who correlated the influence of BCG therapy on growth inhibition of transplanted polyoma rat tumours with increased levels of cytotoxic lymphocytes. This effect was observed when BCG was given intracutaneously before or at the time of tumour implantation. Enhancement of tumour growth rather than suppression was observed when BCG was given at the time when palpable tumour nodules were present, and this correlated with an increased serum blocking activity and no great change in the level of cell mediated immunity. Since *in vitro* correlations of the immune response elicited by sarcoma Mc7–BCG mixed inocula have not yet been analysed, discussion of the role of blocking serum factors is not appropriate. The present studies do indicate, however, that active immunotherapy with BCG vaccine in admixture with tumour cells is feasible. These studies emphasize again that BCG in contact with tumour cells is an essential requirement. Even with highly immunogenic tumours, however, the conditions of treatment must be carefully controlled if a positive response is to be achieved.

This work was supported by the Cancer Research Campaign. We thank Glaxo Research Ltd who kindly supplied the BCG vaccine.

### REFERENCES

BALDWIN, R. W. & PIMM, M. V. (1971) Influence of BCG Infection on Growth of 3-methylcholanthrene-induced Rat Sarcomas. *Eur. J. clin. biol. Res.*, **16**, 875.

BALDWIN, R. W. & PIMM, M. V. (1973*a*) BCG Immunotherapy of Local Subcutaneous Growths and Post-surgical Pulmonary Metastases of a Transplanted Rat Epithelioma of Spontaneous Origin. *Int. J. Cancer.* In the press.

BALDWIN, R. W. & PIMM, M. V. (1973*b*) BCG Immunotherapy of Pulmonary Growths from Intravenously Transferred Rat Tumour Cells. *Br. J. Cancer*, **27**, 48.

BALDWIN, R. W., PRICE, M. R. & ROBINS, R. A. (1973) Significance of Serum Factors Modifying Cellular Immune Responses to Growing Tumours. *Br. J. Cancer.* In the press.

BANSAL, S. C. & SJÖGREN, H. O. (1973) Effects of BCG on Various Facets of the Immune Response against Polyoma Tumors in Rats. *Int. J. Cancer*, **11**, 162.

BARTLETT, G. L. & ZBAR, B. (1972) Tumor-specific Vaccine containing *Mycobacterium bovis* and Tumor Cells: Safety and Efficacy. *J. natn. Cancer Inst.*, **48**, 1709.

BARTLETT, G. L., ZBAR, B. & RAPP, H. J. (1972) Suppression of Murine Tumor Growth by Immune Reaction to the Bacillus Calmette–Guérin Strain of *Mycobacterium bovis*. *J. natn. Cancer Inst.*, **48**, 245.

CURRIE, G. A. & BAGSHAWE, K. D. (1970) Active Immunotherapy with *Corynebacterium parvum* and Chemotherapy in Murine Fibrosarcomas. *Br. med. J.*, i, 541.

HELLSTRÖM, I., HELLSTRÖM, K. E., SJÖGREN, H. O. & WARNER, G. A. (1971) Demonstration of Cell-mediated Immunity to Human Neoplasms of Various Histological Types. *Int. J. Cancer*, **7**, 1.

MATHÉ, G., POUILLART, P. & LAPEYRAQUE, R. (1969) Active Immunotherapy of L1210 Leukaemia Applied after the Graft of Tumour Cells. *Br. J. Cancer*, **23**, 814.

PARR, I. (1972) Response of Syngeneic Murine Lymphomata to Immunotherapy in Relation to the Antigenicity of the Tumour. *Br. J. Cancer*, **26**, 174.

WOODRUFF, M. F. A. & BOAK, J. L. (1966) Inhibitory Effects of Injection of *C. parvum* on the Growth of Tumour Transplants in Isogeneic Hosts. *Br. J. Cancer*, **20**, 345.

ZBAR, B., BERNSTEIN, I. D. & RAPP, H. J. (1971) Suppression of Tumor Growth at the Site of Infection with Living Bacillus Calmette–Guérin. *J. natn. Cancer Inst.*, **46**, 831.

ZBAR, B., BERNSTEIN, I. D., BARTLETT, G. L., HANNA, M. G. & RAPP, H. J. (1972) Immunotherapy of Cancer: Regression of Intradermal Tumors and Prevention of Growth of Lymph Node Metastases after Intralesional Injection of Living *Mycobacterium bovis*. *J. natn. Cancer Inst.*, **49**, 119.